# Supplementary material for: Revisiting the “satisfaction of spatial restraints” approach of MODELLER for protein homology modeling
Source: PLoS Comput Biol. 2019 Dec 17;15(12):e1007219. doi: 10.1371/journal.pcbi.1007219 (PMC6938380; doi:10.1371/journal.pcbi.1007219)
Supplement: S1 Table — Note how by default the objective function does not include any “physical” attractive term between non-bonded atoms (Lennard-Jones and Coulomb potential terms from CHARMM22 [1] are missing). The only attractive terms in the objective function are homology-derived distance restraints (see S2 Table). (PDF) [file pcbi.1007219.s001.pdf]

S1 Table. Physical terms of the MODELLER objective function. Note how by default the objective function does not include any “physical” attractive term between non-bonded atoms (Lennard-Jones and Coulomb potential terms from CHARMM22 [1] are missing). The only attractive terms in the objective function are homology-derived distance restraints (see **S2 Table**).

	**Term**	**MODELLER code**[a]	**Objective function form**[b]
**Physical terms**	CHARMM22 covalent bond length potential	1	harmonic potential
	CHARMM22 bond angle potential	2	harmonic potential
	CHARMM22 stereochemical cosine torsion potential	3	cosine potential
	CHARMM22 stereochemical improper torsion potential	4	harmonic potential
	Soft sphere overlap	5	harmonic lower bound potential
**MODELLER restraints**	ω angle restraints	13	harmonic potential
	Disulfide distance restraints	18	harmonic potential
	Disulfide angle restraints	19	harmonic potential
	Disulfide dihedral angle restraints	20	cosine potential

- a: code used by MODELLER for referring to terms of the objective function.
- b: form of the objective function associated to a term.

References

[1] Brooks BR, Brooks CL, Mackerell AD, Nilsson L, Petrella RJ, Roux B, et al. CHARMM: the biomolecular simulation program. *J Comput Chem.* 2009;30: 1545–1614. doi:10.1002/jcc.21287
