# Supplementary material for: Revisiting the “satisfaction of spatial restraints” approach of MODELLER for protein homology modeling
Source: PLoS Comput Biol. 2019 Dec 17;15(12):e1007219. doi: 10.1371/journal.pcbi.1007219 (PMC6938380; doi:10.1371/journal.pcbi.1007219)
Supplement: S2 Table — (PDF) [file pcbi.1007219.s002.pdf]

S2 Table. Homology-derived terms of the MODELLER objective function.

	Homology-derived spatial restraint[a]	MODELLER code[b]	d_{max} (Å)[c]	r_{min}, r_{max}[d]	*pdf*[e]	Average number of restraints per model[f]
**Homology-derived distance restraints (HDDR)**	Cα-Cα	9	14.0	2, ∞	Gaussian	2968.33
	NO	10	11.0	2, ∞	Gaussian	3084.76
	SCMC	23	5.5	1, 2	Gaussian	1632.12
	SCSC	26	5.0	2, ∞	Gaussian	606.56
	Total distance restraints	-	-	-	-	8291.77
**Homology-derived dihedral angle restraints (HDAR)**	χ_1	14	-	-	weighted sum of Gaussians	146.95
	χ_2	15	-	-	weighted sum of Gaussians	111.56
	χ_3	16	-	-	weighted sum of Gaussians	48.86
	χ_4	17	-	-	weighted sum of Gaussians	18.81
	φ and ψ	25	-	-	weighted sum of bivariate Gaussians	170.98
	Total dihedral restraints	-	-	-	-	497.16

- a: names of the homology-derived spatial restraints groups of MODELLER. Cα-Cα (Carbon α distance restraints); NO (main chain nitrogen and oxygen distance restraints); SCMC (side chain - main chain distance restraints); SCSC (side chain - side chain distance restraints).
- b: code used by MODELLER for referring to terms of the objective function.
- c: MODELLER will build a restraint of this group between two atoms only if the equivalent distance in the template is smaller than d_{max}.
- d: MODELLER will build a restraint of this group between two atoms only if the difference between the numerical indices of their residues is >= r_{min} and < r_{max}.
- e: probability density function associated to a restraint.
- f: average number of homology-derived spatial restraints per model in the AS set. Note that the average length of the target proteins of this set is 172.98 residues.
