# Supplementary material for: Revisiting the “satisfaction of spatial restraints” approach of MODELLER for protein homology modeling
Source: PLoS Comput Biol. 2019 Dec 17;15(12):e1007219. doi: 10.1371/journal.pcbi.1007219 (PMC6938380; doi:10.1371/journal.pcbi.1007219)
Supplement: S3 Table — See Table 1 in the main text for the description of contents, columns and most modeling strategies names. (PDF) [file pcbi.1007219.s003.pdf]

S3 Table. 3D modeling qualities of the AS single-template models built with different modeling strategies. See **Table 1** in the main text for the description of contents, columns and most modeling strategies names.

**Strategy**	**GDT-HA**	**GDT-HA p-value**	**lDDT**	**LDDT p-value**	**MolProbity score**	**MolProbity score p-value**
MODELLER	0.6014 (-)	-	0.6563 (-)	-	3.0104 (-)	-
OPTIMAL	0.6377 (+6.0%)	1.2e-38	0.6842 (+4.2%)	1.1e-38	3.0311 (+0.7%)	9.5e-4
MODELLER-SLOW	0.6036 (+0.4%)	6.4e-6	0.6594 (+0.5%)	3.6e-28	2.8512 (-5.3%)	2.3e-37
OPTIMAL-SLOW	0.6377 (+6.0%)	1.2e-38	0.6853 (+4.4%)	1.1e-38	2.9039 (-3.5%)	2.2e-17
MODELLER-TMalign	0.6383 (+6.1%)	1.1e-31	0.6951 (+5.9%)	6.7e-29	3.0411 (+1.0%)	4.1e-2
OPTIMAL-TMalign	0.6805 (+13.2%)	1.1e-38	0.7259 (+10.6%)	1.9e-37	3.0870 (+2.5%)	5.1e-7
MODELLER-DOPE-0.5	0.6089 (+1.3%)	2.2e-17	0.6692 (+2.0%)	1.9e-36	2.1138 (-29.8%)	1.1e-38
MODELLER-SLOW-DOPE-0.5	0.6112 (+1.6%)	1.6e-21	0.6746 (+2.8%)	1.9e-38	2.0344 (-32.4%)	1.1e-38
MODELLER-DOPE-3.5	0.5631 (-6.4%)	7.8e-28	0.6397 (-2.5%)	1.9e-15	2.9977 (-0.4%)	2.4e-1
OPTIMAL-DOPE-0.5	0.6549 (+8.9%)	1.1e-38	0.7029 (+7.1%)	1.1e-38	2.2960 (-23.7%)	1.1e-38
OPTIMAL-DOPE-3.5	0.6885 (+14.5%)	1.1e-38	0.7158 (+9.1%)	1.1e-38	2.6280 (-12.7%)	1.8e-36
MODELLER-DFIRE-0.5[a]	0.6086 (+1.2%)	3.1e-16	0.6656 (+1.4%)	1.4e-29	2.0801 (-30.9%)	1.1e-38
MODELLER-SLOW-DFIRE-0.5	0.6112 (+1.6%)	7.1e-22	0.6701 (+2.1%)	5.9e-37	1.9818 (-34.2%)	1.1e-38
MODELLER-DFIRE-3.5	0.5698 (-5.2%)	4.6e-24	0.6365 (-3.0%)	2.8e-20	2.7926 (-7.2%)	1.5e-29
OPTIMAL-DFIRE-0.5	0.6557 (+9.0%)	1.1e-38	0.7013 (+6.9%)	1.1e-38	2.2843 (-24.1%)	1.1e-38
OPTIMAL-DFIRE-3.5	0.6907 (+14.9%)	1.1e-38	0.7138 (+8.8%)	1.1e-38	2.4929 (-17.2%)	1.5e-38

- a: the “DFIRE-X.X” suffix indicates the use of DFIRE with a w_{SP} of X.X.
