# Supplementary material for: Revisiting the “satisfaction of spatial restraints” approach of MODELLER for protein homology modeling
Source: PLoS Comput Biol. 2019 Dec 17;15(12):e1007219. doi: 10.1371/journal.pcbi.1007219 (PMC6938380; doi:10.1371/journal.pcbi.1007219)
Supplement: S4 Table — See Tables 1 and 2 in the main text and S3 Table for the description of contents, columns and most modeling strategies names. (PDF) [file pcbi.1007219.s004.pdf]

S4 Table. 3D modeling qualities of the AM multiple-templates models built with different modeling strategies. See **Table 1** and **2** in the main text and **S3 Table** for the description of contents, columns and most modeling strategies names.

Strategy	GDT-HA	GDT-HA p-value	lDDT	LDDT p-value	MolProbity score	MolProbity score p-value
MODELLER	0.6287 (-)	-	0.6819 (-)	-	3.0725 (-)	-
OPTIMAL	0.8733 (+38.9%)	9.8e-21	0.8106 (+18.9%)	1.9e-20	3.1478 (+2.4%)	9.8e-1
MODELLER-SLOW	0.6310 (+0.4%)	3.0e-3	0.6850 (+0.5%)	3.8e-12	2.9143 (-5.2%)	2.4e-20
OPTIMAL-SLOW	0.8747 (+39.1%)	9.3e-21	0.8133 (+19.3%)	1.9e-20	3.0475 (-0.8%)	1.5e-4
OPTIMAL-U	0.7438 (+18.3%)	3.1e-13	0.7427 (+8.9%)	2.5e-13	3.1744 (+3.3%)	3.1e-1
MODELLER-ST	0.6168 (-1.9%)	9.0e-5	0.6683 (-2.0%)	1.7e-7	3.0231 (-1.6%)	4.6e-2
OPTIMAL-ST	0.6557 (+4.3%)	1.1e-11	0.6986 (+2.5%)	8.9e-11	3.0398 (-1.1%)	8.3e-1
MODELLER-TMalign	0.6645 (+5.7%)	6.4e-17	0.7165 (+5.1%)	8.4e-17	3.0529 (-0.6%)	1.7e-1
OPTIMAL-TMalign	0.9222 (+46.7%)	6.3e-21	0.8498 (+24.6%)	8.4e-21	3.1044 (+1.0%)	1.3e-1
MODELLER-DOPE-0.5	0.6327 (+0.6%)	1.5e-3	0.6926 (+1.6%)	5.7e-17	2.2086 (-28.1%)	4.2e-21
MODELLER-SLOW-DOPE-0.5	0.6347 (+1.0%)	7.6e-05	0.6971 (+2.2%)	4.1e-19	2.1152 (-31.2%)	4.2e-21
MODELLLER-DOPE-3.5	0.5646 (-10.2%)	4.3e-18	0.6453 (-5.4%)	7.5e-17	3.1267 (+1.8%)	2.4e-2
OPTIMAL-DOPE-0.5	0.8736 (+39.0%)	1.0e-20	0.8229 (+20.7%)	1.4e-20	2.5635 (-16.6%)	5.7e-13
OPTIMAL-DOPE-3.5	0.8519 (+35.5%)	1.1e-20	0.8061 (+18.2%)	1.5e-20	2.7520 (-10.4%)	1.4e-11
MODELLER-DFIRE-0.5	0.6322 (+0.6%)	8.0e-3	0.6892 (+1.1%)	6.0e-13	2.1672 (-29.5%)	4.2e-21
MODELLER-SLOW-DFIRE-0.5	0.6353 (+1.1%)	8.8e-06	0.6934 (+1.7%)	8.5e-18	2.0573 (-33.0%)	4.2e-21
MODELLER-DFIRE-3.5	0.5735 (-8.8%)	1.4e-16	0.6445 (-5.5%)	2.8e-17	2.9027 (-5.5%)	1.7e-11
OPTIMAL-DFIRE-0.5	0.8740 (+39.0%)	1.0e-20	0.8209 (+20.4%)	1.5e-20	2.5617 (-16.6%)	5.9e-13
OPTIMAL-DFIRE-3.5	0.8540 (+35.8%)	1.1e-20	0.8051 (+18.1%)	1.7e-20	2.6626 (-13.3%)	8.0e-13
