# Supplementary material for: Revisiting the “satisfaction of spatial restraints” approach of MODELLER for protein homology modeling
Source: PLoS Comput Biol. 2019 Dec 17;15(12):e1007219. doi: 10.1371/journal.pcbi.1007219 (PMC6938380; doi:10.1371/journal.pcbi.1007219)
Supplement: S1 Fig — (A) SeqId histogram of the pairwise target-template alignments in the AS models obtained using TM-align and HHalign. (B) Target coverage histograms of the same alignments. (C) Chain length histograms of the 225 AS targets, the 118 AM targets and all the 472 template chains of the analysis set. (D) CATH classes frequencies of the AS and AM targets compared to those in the entire CATH 4.2.0 database [1]. (PDF) [file pcbi.1007219.s005.pdf]

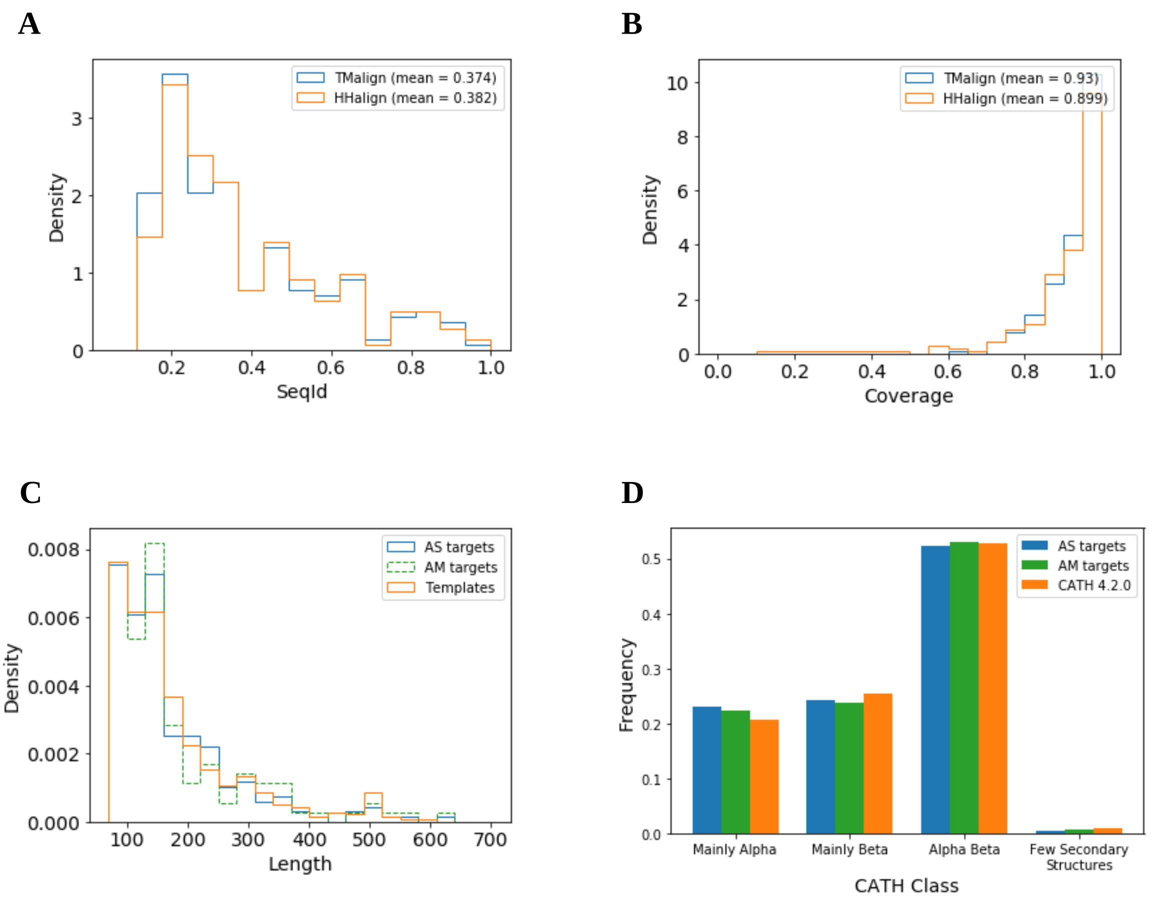

S1 Fig. Properties of the analysis set. (A) SeqId histogram of the pairwise target-template alignments in the AS models obtained using TM-align and HHalign. (B) Target coverage histograms of the same alignments. (C) Chain length histograms of the 225 AS targets, the 118 AM targets and all the 472 template chains of the analysis set. (D) CATH classes frequencies of the AS and AM targets compared to those in the entire CATH 4.2.0 database [1].

References

[1] Dawson NL, Lewis TE, Das S, Lees JG, Lee D, Ashford P, et al. CATH: an expanded resource to predict protein function through structure and sequence. *Nucleic Acids Res.* 2017;45: D289–D295. doi:10.1093/nar/gkw1098
