# Supplementary material for: Revisiting the “satisfaction of spatial restraints” approach of MODELLER for protein homology modeling
Source: PLoS Comput Biol. 2019 Dec 17;15(12):e1007219. doi: 10.1371/journal.pcbi.1007219 (PMC6938380; doi:10.1371/journal.pcbi.1007219)
Supplement: S2 Fig — (A) to (E) the Cα-Cα |Δdn| values of the 5jwo_chain_B (target) - 1thx_chain_A (template) pair were perturbed to various PCCSEL levels using the perturbation scheme described in the “Methods” section of the main text. The observed PCCs between the perturbed and the original |Δdn| values are reported. (F) Distributions of the original |Δdn| values and three perturbed values lists shown in previous figures. The mean values of the lists are reported in brackets. Thanks to the use of Laplace distributions for extracting random errors, the perturbed values are distributed approximately as exponentials, which resemble the original |Δdn| distribution. (G) and (H) average PCCMODEL values of the AS and AM models in |Δdn| perturbation experiments plotted as a function of PCCSEL. On average, each PCCSEL value allows to obtain almost exactly the desired level of perturbation (quantified as PCCMODEL). Data for the four HDDRs groups of MODELLER is shown. (G) AS models. (H) AM models. (PDF) [file pcbi.1007219.s006.pdf]

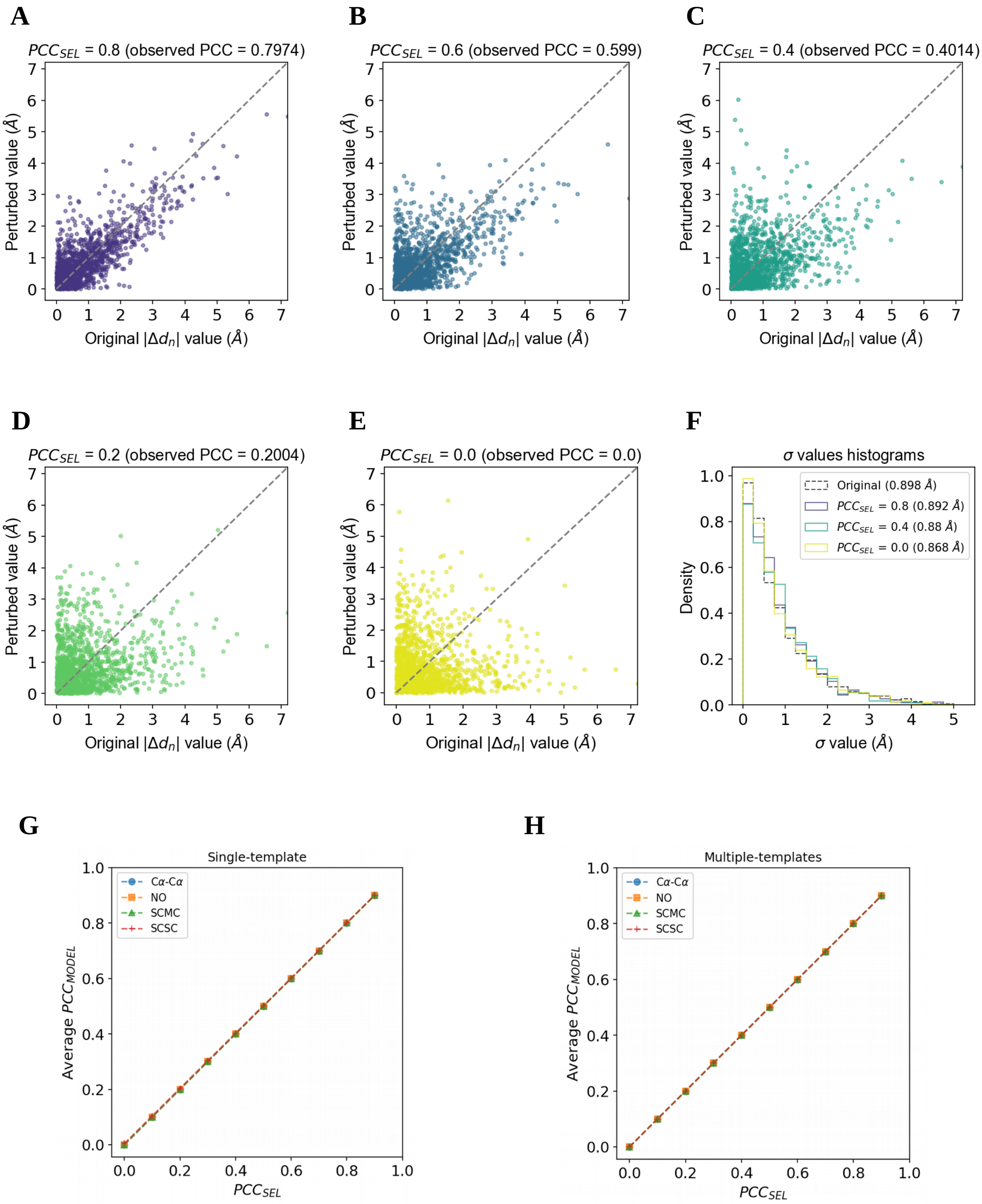

S2 Fig. Details of the |Δd_n| perturbation scheme. (A) to (E) the Cα-Cα |Δd_n| values of the *5jwo_chain_B* (target) - *1thx_chain_A* (template) pair were perturbed to various PCC_{SEL} levels using the perturbation scheme described in the "Methods" section of the main text. The observed PCCs between the perturbed and the original |Δd_n| values are reported. (F) Distributions of the original |Δd_n| values and three perturbed values lists shown in previous figures. The mean values of the lists are reported in brackets. Thanks to the use of Laplace distributions for extracting random errors, the perturbed values are distributed approximately

as exponentials, which resemble the original $|\Delta d_n|$ distribution. (G) and (H) average PCC_{MODEL} values of the AS and AM models in $|\Delta d_n|$ perturbation experiments plotted as a function of PCC_{SEL}. On average, each PCC_{SEL} value allows to obtain almost exactly the desired level of perturbation (quantified as PCC_{MODEL}). Data for the four HDDRs groups of MODELLER is shown. (G) AS models. (H) AM models.
