# Supplementary material for: Revisiting the “satisfaction of spatial restraints” approach of MODELLER for protein homology modeling
Source: PLoS Comput Biol. 2019 Dec 17;15(12):e1007219. doi: 10.1371/journal.pcbi.1007219 (PMC6938380; doi:10.1371/journal.pcbi.1007219)
Supplement: S3 Fig — (A) Forms of the 12561 terms of DOPE [1]. Each term is associated to a couple of heavy atom types from the 20 standard residues. Irrespective of the atom types, all the functions start to acquire a flat shape above the 8.0 Å threshold. (B) Confrontation of DOPE and DFIRE [2] terms. An hexbin density plot compares 364269 data points from all the 12561 terms of DOPE (x-axis) and DFIRE (y-axis) (each term has 29 points, which report the score of the potential in a linear space from 0.75 to 14.75 Å). The scores of the two potentials are highly correlated (Pearson correlation coefficient = 0.99). (PDF) [file pcbi.1007219.s007.pdf]

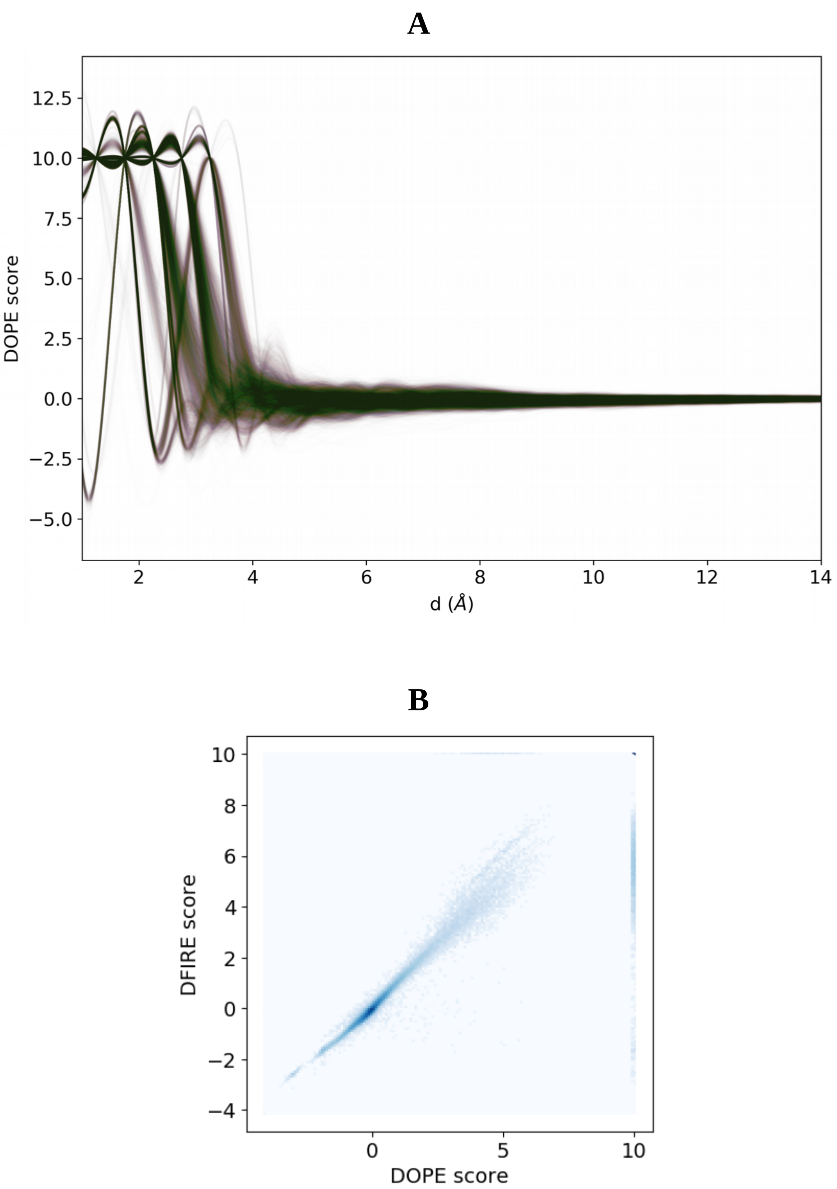

S3 Fig. Analysis of the terms of the DOPE and DFIRE potentials. (A) Forms of the 12561 terms of DOPE [1]. Each term is associated to a couple of heavy atom types from the 20 standard residues. Irrespective of the atom types, all the functions start to acquire a flat shape above the 8.0 Å threshold. (B) Confrontation of DOPE and DFIRE [2] terms. An hexbin density plot compares 364269 data points from all the 12561 terms of DOPE (x-axis) and DFIRE (y-axis) (each term has 29 points, which report the score of the potential in a linear space from 0.75 to 14.75 Å). The scores of the two potentials are highly correlated (Pearson correlation coefficient = 0.99).

References

[1] Shen M-Y, Sali A. Statistical potential for assessment and prediction of protein structures. Protein Sci. 2006;15: 2507–2524. doi:10.1110/ps.062416606

[2] Zhou H, Zhou Y. Distance-scaled, finite ideal-gas reference state improves structure-derived potentials of mean force for structure selection and stability prediction. Protein Sci. 2002;11: 2714–2726. doi:10.1110/ps.0217002
