# Supplementary material for: Revisiting the “satisfaction of spatial restraints” approach of MODELLER for protein homology modeling
Source: PLoS Comput Biol. 2019 Dec 17;15(12):e1007219. doi: 10.1371/journal.pcbi.1007219 (PMC6938380; doi:10.1371/journal.pcbi.1007219)
Supplement: S4 Fig — The x-axis reports the SeqId between the target and template sequences in TM-align alignments. The y-axis reports the accuracy of the corresponding HHalign alignment. The accuracy is computed as the ratio Hm/Tm, where Tm is the total number of matches in the TM-align alignment and Hm is the number of “correct” matches in HHalign alingments (that is, those HHalign matches which are also found in the TM-align alignment). The average accuracy is 0.87. (PDF) [file pcbi.1007219.s008.pdf]

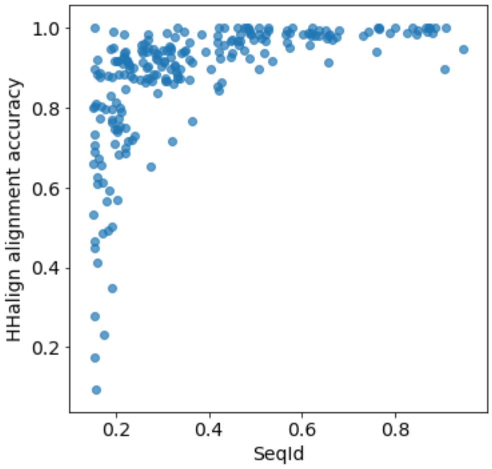

S4 Fig. Accuracy of the pairwise target-template HHalign alignments of the AS models. The x-axis reports the SeqId between the target and template sequences in TM-align alignments. The y-axis reports the accuracy of the corresponding HHalign alignment. The accuracy is computed as the ratio H_m/T_m, where T_m is the total number of matches in the TM-align alignment and H_m is the number of "correct" matches in HHalign alingments (that is, those HHalign matches which are also found in the TM-align alignment). The average accuracy is 0.87.
