# Supplementary material for: Revisiting the “satisfaction of spatial restraints” approach of MODELLER for protein homology modeling
Source: PLoS Comput Biol. 2019 Dec 17;15(12):e1007219. doi: 10.1371/journal.pcbi.1007219 (PMC6938380; doi:10.1371/journal.pcbi.1007219)
Supplement: S5 Fig — The horizontal dashed lines correspond to the scores obtained when modeling with MODELLER-generated (blue color) or optimal (orange) HDDRs without the use of statistical potentials. (A) to (C) quality scores of the AS models. (D) to (F) quality scores of the AM models. (PDF) [file pcbi.1007219.s009.pdf]

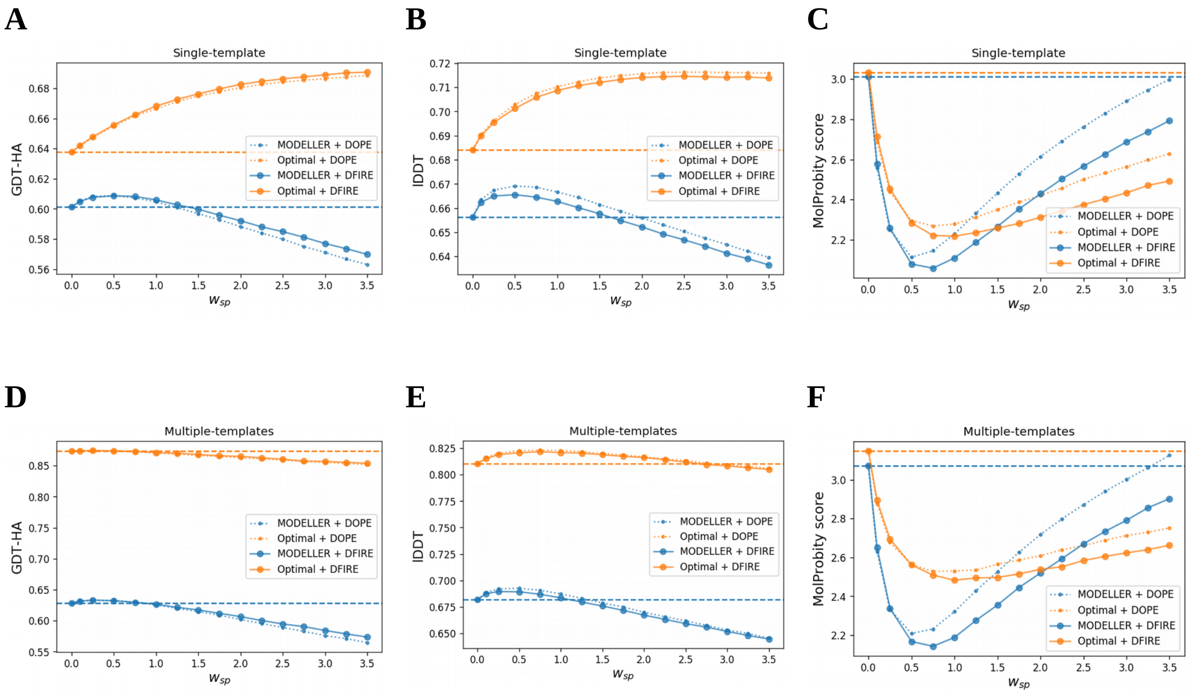

S5 Fig. Average quality scores of the analysis set models as a function of the w_{sp} value with which the DFIRE or DOPE statistical potentials have been included in the objective function of MODELLER. The horizontal dashed lines correspond to the scores obtained when modeling with MODELLER-generated (blue color) or optimal (orange) HDDRs without the use of statistical potentials. (A) to (C) quality scores of the AS models. (D) to (F) quality scores of the AM models.
