# Supplementary material for: Revisiting the “satisfaction of spatial restraints” approach of MODELLER for protein homology modeling
Source: PLoS Comput Biol. 2019 Dec 17;15(12):e1007219. doi: 10.1371/journal.pcbi.1007219 (PMC6938380; doi:10.1371/journal.pcbi.1007219)
Supplement: S1 Text — (PDF) [file pcbi.1007219.s010.pdf]

S1 Text. Description of the GDT-HA and lDDT metrics for model quality evaluation.

GDT-HA. To evaluate the backbone quality of the 3D models we used the global distance test - high accuracy (GDT-HA) metric, one of the most frequently used in literature and in CASP experiments [1]. Once a 3D model is superposed to the target experimentally-determined structure, GDT-HA is computed as:

$$GDT\text{-}HA = (p_{0.5} + p_{1.0} + p_{2.0} + p_{4.0})/4$$

where p_d is the fraction of Cα atoms of the 3D model found at a distance of less than d Å of their equivalent atoms in the experimentally-determined structure. GDT-HA values range from 0 to 1 (with 1 corresponding to a maximum of accuracy). In order to compute GDT-HA scores, we used the TM-score program available at:

https://zhanglab.ccmb.med.umich.edu/TM-score.

lDDT. In order to evaluate the quality of local structures and side chains of the models we used the local distance difference test (lDDT) metric [2]. This metric is computed by considering all pairs of heavy atoms in the target experimentally-determined structure at a distance closer than 15.0 Å and not belonging to the same residue. These interatomic distances are compared with the equivalent ones in the 3D model. A distance in the model is presumed to be correctly modeled if the difference between its value and that of its equivalent distance in the target is below a specific cutoff. The lDDT score is computed as:

$$lDDT = (f_{0.5} + f_{1.0} + f_{2.0} + f_{4.0})/4$$

where f_d is the fraction of distances being correctly modeled by considering a cutoff value of f Å. lDDT values range from 0 to 1 (with 1 corresponding to a maximum of accuracy). In order to compute this metric, we used the lDDT program available at:

https://swissmodel.expasy.org/lddt.

References

[1] Kryshtafovych A, Monastyrskyy B, Fidelis K, Moult J, Schwede T, Tramontano A. Evaluation of the template-based modeling in CASP12. *Proteins.* 2018;86 Suppl 1: 321–334. doi:10.1002/prot.25425

[2] Mariani V, Biasini M, Barbato A, Schwede T. lDDT: a local superposition-free score for comparing protein structures and models using distance difference tests. *Bioinformatics.* 2013;29: 2722–2728. doi:10.1093/bioinformatics/btt473
