# Supplementary material for: Revisiting the “satisfaction of spatial restraints” approach of MODELLER for protein homology modeling
Source: PLoS Comput Biol. 2019 Dec 17;15(12):e1007219. doi: 10.1371/journal.pcbi.1007219 (PMC6938380; doi:10.1371/journal.pcbi.1007219)
Supplement: S2 Text — (PDF) [file pcbi.1007219.s011.pdf]

S2 Text. Obtaining optimal parameters for single-template HDDRs.

The use of optimal homology-derived distance restraints (HDDRs) on single-template modeling has been shown to improve 3D modeling quality [1]. In this section we show how a theoretical model can be used explain this behavior. When building a Gaussian HDDR for a couple of atoms *i* and *j* in a model, MODELLER finds in the template the equivalent atoms *k* and *l* having a distance d_t. The distance d_m between *i* and *j* will be restrained using a Gaussian *pdf* of the form:

$$f(d_m)=\frac{1}{\sigma\sqrt{2\pi}}e^{-\frac{(d_m-d_t)^2}{2\sigma^2}} \quad .$$

Different *σ* values change the likelihood of obtaining a model in which d_m is equal to d_n, the distance observed in the experimentally-determined target structure. The *σ* which maximizes this likelihood can be found in the following way. When d_m is equal to d_n the value of the *pdf* is:

$$f(d_m=d_n)=\frac{1}{\sigma\sqrt{2\pi}}e^{-\frac{(d_n-d_t)^2}{2\sigma^2}}=\frac{1}{\sigma\sqrt{2\pi}}e^{-\frac{\Delta d_n^2}{2\sigma^2}}$$

where

$$\Delta d_n=d_n-d_t \quad .$$

As shown in **Fig A** in **S2 Text**, this value will change according to the *σ* of the *pdf*. Therefore $f(d_m = d_n)$ can be expressed as a function *g* of *σ*:

$$f(d_m=d_n)=g(\sigma)=\frac{1}{\sigma\sqrt{2\pi}}e^{-\frac{\Delta d_n^2}{2\sigma^2}}$$

the value of *σ* which maximizes *g(σ)* can be easily found by derivating this function and putting the derivative equal to zero. The derivative of this function is:

$$\frac{d}{d\sigma}[g(\sigma)]=e^{-\frac{\Delta d_n^2}{2\sigma^2}}\left(\frac{\Delta d_n^2}{\sigma^4\sqrt{2\pi}}-\frac{1}{\sigma^2\sqrt{2\pi}}\right)$$

and by putting it to zero, we see that:

$$\frac{\Delta d_n^2}{\sigma^4\sqrt{2\pi}}=\frac{1}{\sigma^2\sqrt{2\pi}}$$

and finally by solving this equation for *σ* we find that:

$$\sigma=|\Delta d_n|=|d_n-d_t|$$

therefore the *σ* value which will maximize the *pdf* (and minimize the MODELLER objective function) at $d_m = d_n$ is exactly $|\Delta d_n|$ (see also **Fig B** in **S2 Text**). Since in MODELLER each *pdf* is independent from the others, the set of *σ* values which will maximize the total *molpdf* when each d_m is equal to its corresponding d_n, is the same one which will maximize each *pdf* independently. Therefore $|\Delta d_n|$ values are theoretically the optimal *σ* values.

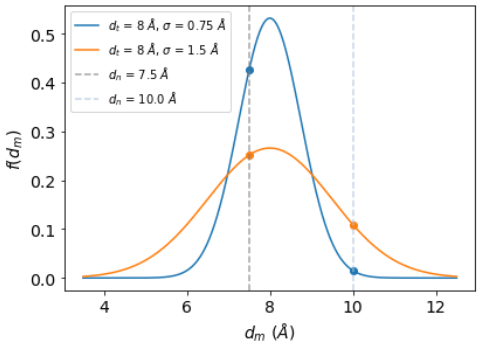

Fig A. Relationship between the σ value of a Gaussian HDDR and the likelihood of building a model with a $d_m = d_n$. The *pdfs* for two HDDRs with the same d_t (8.0 Å) but with different σ values are compared. In this example, if the native distance d_n is 7.5 Å, the narrower *pdf* (with a σ of 0.75 Å, colored in blue) has an higher likelihood of reproducing d_n. If d_n is 10.0 Å, the wider *pdf* (with a σ of 1.5 Å, colored in orange) has an higher likelihood of reproducing it. Figure adapted from [2].

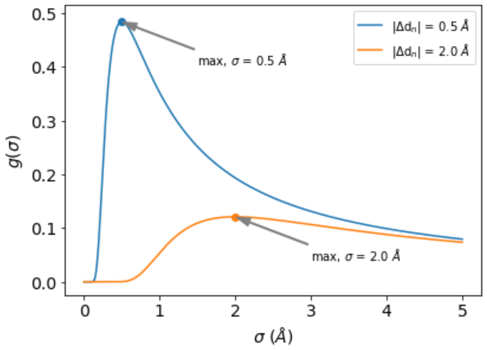

Fig B. The likelihood of producing a model in which $d_m = d_n$ is a function of the σ value of the restraint. This likelihood is maximized when $\sigma = |\Delta d_n|$, as shown for the two restraints described in **Fig A** in **S2 Text**.

References

[1] Lee J, Lee K, Joung I, Joo K, Brooks BR, Lee J. Sigma-RF: prediction of the variability of spatial restraints in template-based modeling by random forest. *BMC Bioinformatics*. 2015;16: 94. doi:10.1186/s12859-015-0526-z

[2] Thompson J, Baker D. Incorporation of evolutionary information into Rosetta comparative modeling. *Proteins*. 2011;79: 2380–2388. doi:10.1002/prot.23046
