# Supplementary material for: Revisiting the “satisfaction of spatial restraints” approach of MODELLER for protein homology modeling
Source: PLoS Comput Biol. 2019 Dec 17;15(12):e1007219. doi: 10.1371/journal.pcbi.1007219 (PMC6938380; doi:10.1371/journal.pcbi.1007219)
Supplement: S3 Text — (PDF) [file pcbi.1007219.s012.pdf]

S3 Text. Obtaining optimal parameters for multiple-template HDDRs.

In this section we show how to obtain the optimal parameters of homology-derived distance restraints in multiple-template modeling. When using *U* templates to restrain a distance d_m in a model, MODELLER employs as a *pdf* the following weighted sum of Gaussians:

$$f(d_m)=\sum_{u=1}^{U} w_u \frac{1}{\sigma_u\sqrt{2\pi}} e^{-\frac{(d_m-d_{t,u})^2}{2\sigma_u^2}}$$

where *u* is the index of the template, w_u is a template-specific weight, $d_{t,u}$ and σ_u are the distance observed the *u*-th template and its σ value respectively. The sum of all the weights is 1:

$$\sum_{u=1}^{U} w_u = 1 \quad .$$

Optimal multiple-template restraints should contain an optimal set σ_u values and w_u weights. The goal is to find the combination of parameters that maximizes the value of the *pdf* when $d_m = d_n$ (where d_n is the distance observed in the target native structure). Let us consider a case with *U* templates T_1, T_2, T_3, …, T_U. If the templates were to be used independently, each one would have its own Gaussian *pdf*. We can define as N_u the value which the single-template *pdf* from template *u* would assume when $d_m = d_n$ in the following way:

$$N_u=\frac{1}{\sigma_u\sqrt{2\pi}} e^{-\frac{(d_n-d_{t,u})^2}{2\sigma_u^2}} \quad .$$

In our example, let us assume that the template yielding the highest value at $d_m = d_n$ is T_1 (see **Fig A** in **S3 Text**). If the templates were to be used in multiple-template modeling, then $f(d_m = d_n)$ would be:

$$f(d_m=d_n)=\sum_{u=1}^{U} w_u N_u \quad .$$

This expression can be written by separating the contribution of T_1 and by incorporating the contributions from all other templates in a single term to yield:

$$f(d_m=d_n)=w_1 N_1+\sum_{u=2}^{U} w_u N_u$$

and since:

$$w_1=1-\sum_{u=2}^{U} w_u$$

the previous expression can be rewritten as:

$$f(d_m=d_n)=\left(1-\sum_{u=2}^{U} w_u\right) N_1+\sum_{u=2}^{U} w_u N_u=N_1-\sum_{u=2}^{U} w_u N_1+\sum_{u=2}^{U} w_u N_u$$

and so we find that:

$$f(d_m = d_n) = N_1 - \sum_{u=2}^{U} w_u (N_1 - N_u)$$

and since N_1 is higher than any other N_u, in order to maximize $f(d_m = d_n)$, all the w_u with the exception of w_1 should be set to 0, while w_1 should be set exactly to 1. Since in single-template modeling the σ value which maximizes a Gaussian *pdf* at $d_m = d_n$ is $|\Delta d_n|$ (see **S2 Text**), in order to maximize N_1 the σ of T_1 must be its $|\Delta d_n|$ value.

To summarize, for multiple-template modeling, the optimal σ values are again $|\Delta d_n|$ values and the optimal weights turn out to be all 0 with exception of the weight of the template with the lowest $|\Delta d_n|$ value, which should be set to 1.

We name this template-weighting scheme as the “only-lowest” (OL) scheme. It can also be applied to restraints having non-optimal σ values (in this case the template with the lowest σ value will receive a weight of 1). The OL scheme is an extreme case of the template-weighting scheme proposed in [1]. In this scheme, a weight is assigned by:

$$w_u = \frac{\sigma_u^{-k}}{\sum_{v=1}^{U} \sigma_v^{-k}}$$

where σ_u is the σ assigned to template u, v is an index to iterate through all templates and k is a tunable parameter. Here, templates with lower σ values are attributed higher weights. The parameter k determines the extent with which templates with lower σ values dominate over those with higher σ values. A value of $k = 0$ assigns all templates equal weights, while a value of $k \rightarrow \infty$ corresponds to the OL scheme described above.

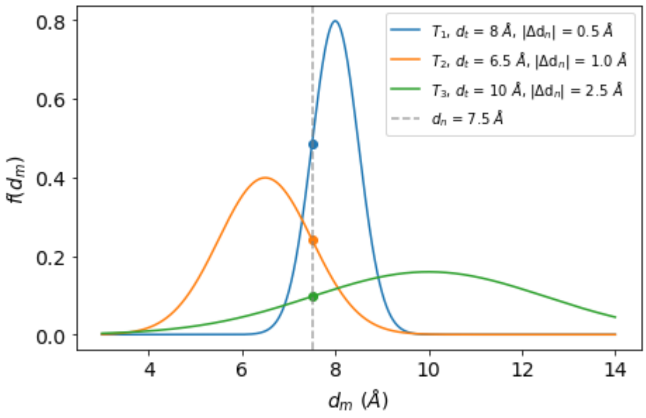

Fig A. Three different single-template Gaussian *pdfs* with optimal σ values are used to model a distance d_m in a 3D model. In this example (where the target native distance d_n is 7.5 Å) the template with the *pdf* having the highest likelihood of reproducing it is T_1 (*pdf* colored in blue) since it has the lowest $|\Delta d_n|$ value (0.5 Å).

References

[1] Thompson J, Baker D. Incorporation of evolutionary information into Rosetta comparative modeling. *Proteins*. 2011;79: 2380–2388. doi:10.1002/prot.23046
