# Supplementary material for: Revisiting the “satisfaction of spatial restraints” approach of MODELLER for protein homology modeling
Source: PLoS Comput Biol. 2019 Dec 17;15(12):e1007219. doi: 10.1371/journal.pcbi.1007219 (PMC6938380; doi:10.1371/journal.pcbi.1007219)
Supplement: S4 Text — (PDF) [file pcbi.1007219.s013.pdf]

S4 Text. Distribution of best templates for multiple-template HDDRs along target sequences.

When performing multiple-template modeling and when using the “only-lowest” (OL) template weighting scheme with optimal σ values, in an homology-derived distance restraint (HDDR) acting on an interatomic distance in a model, the only contribution is that of the “best template” for that distance (that is, the template with the lowest $|\Delta d_n|$ value for that distance). In order to establish how the best templates are distributed throughout the target sequences of the AM set, we proceeded in the following way.

For each restrained residue of a multiple-template model, we considered all the Cα-Cα HDDRs acting on it (here, we only report results obtained for Cα-Cα HDDRs, but similar conclusions can be drawn for other HDDRs groups). In the AM models, the average number of Cα-Cα HDDRs acting on a restrained residue is 43.4. For a target sequence having U templates, the fraction of HDDRs acting on residue i in which template u is the best template can be expressed as:

$$f_{i,u}=\frac{n_{i,u}}{n_{i,tot}} \quad ,$$

where $n_{i,u}$ is the number of HDDRs acting on residue i in which the best template is template u and $n_{i,tot}$ is the total number of HDDRs acting on i (see **Fig C** in **S4 Text**). To quantify whether on a single residue there are templates which are picked more frequently than others or if all templates are picked with similar frequencies, we can define H_i, the “best template Shannon entropy”, for residue i as:

$$H_i=-\sum_{u=1}^{U} f_{i,u}\log_2(f_{i,u}) \quad .$$

This value is equal to 0 if only one template is picked as the best template for all the HDDRs acting on residue i, while it is equal to $-log_2(1/U)$ if all templates are picked with an equal share. The average entropy of a target protein can be expressed as:

$$H_{mean}=\frac{1}{N}\sum_{i=1}^{N} H_i \quad ,$$

where N is the total number of restrained residues of the target. To compare the entropies of targets with different number of templates, the average entropy of a target with U templates can be normalized in the following way:

$$H_{norm}=\frac{H_{mean}}{H_{max}} \quad ,$$

where H_{max} is the maximum theoretical average entropy which a target sequence with the same number of restrained residues and with the same number of templates could acquire (that is, the entropy which would be reached if in all the restrained residues each template was picked with exactly the same frequency):

$$H_{max} = \frac{1}{N}\sum_{i=1}^{N} -\log_2\left(\frac{1}{U}\right) = -\log_2\left(\frac{1}{U}\right) \quad .$$

H_{norm} values range from 0 to 1. Lower H_{norm} values indicate a target protein in which single templates tend to dominate different residues, while higher values indicate a protein in which different templates are effectively used together to optimally model residues.

Fig D in **S4 Text** reports the distribution of the H_{norm} scores of all the 118 AM models and shows that the average H_{norm} value is 0.807. **Fig A** and **B** in **S4 Text** show the representative cases of target *1dk8_chain_A* (with an H_{norm} value of 0.876, being at the 70th percentile of the H_{norm} values of the AM models) and *3m5q_chain_A* (with a H_{norm} value of 0.687, being at the 12th percentile). In the first example, we have three templates with about the same coverage and SeqId with the target sequence. As it can be seen from the $f_{i,u}$ and H_i plots, for most residues, the three templates are picked with similar frequencies (resulting in very few residues with low entropy values) and no template dominates over some extended region (the only exception are the last few C-terminal restrained target residues, which in the alignment are covered only by the *1fqi_chain_A* template, so that only this template can provide HDDRs for them). In the second example, we have five templates, with one template (*4czq_chain_A*) with an higher SeqId with respect to the others. While this template is picked more frequently as the best template, also the other four templates with lower similarity are represented in most residues and no template seems to dominate among these four.

As seen from the H_{norm} histogram, most proteins in the AM set have high H_{norm} values and their situations resemble these two cases. Taken together, this data shows that in the multiple-template models, the contributions of the best templates are typically intertwined throughout the residues of the target sequences.

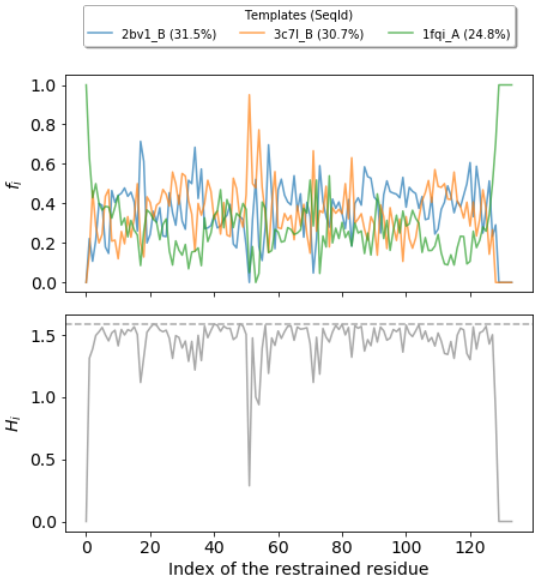

Fig A. $f_{i,u}$ and H_i plots for target *1dk8_chain_A*. The names of the templates (and in brackets their SeqId with the target sequence) are indicated in the legends. The dashed horizontal lines in the H_i plots represent the maximum entropy values, that is, the H_i value which a residue would have if in its HDDRs each template had the same $f_{i,u}$. This value equals to $-log_2(1/U)$ with U being the number of templates of the target.

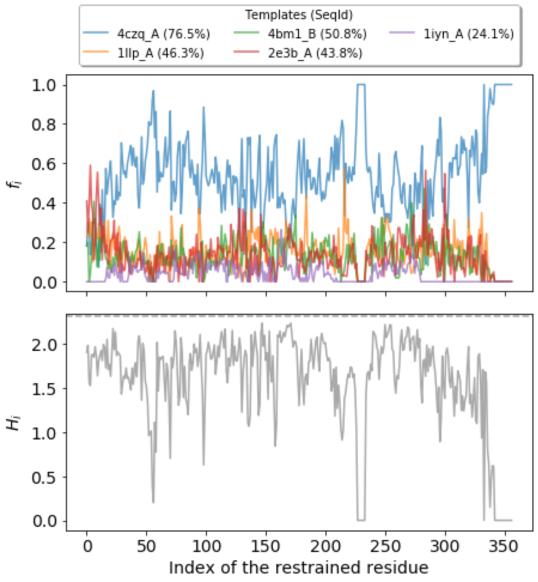

Fig B. $f_{i,u}$ and H_i plots for target *3m5q_chain_A*. See **Fig A** in **S4 Text** for more information.

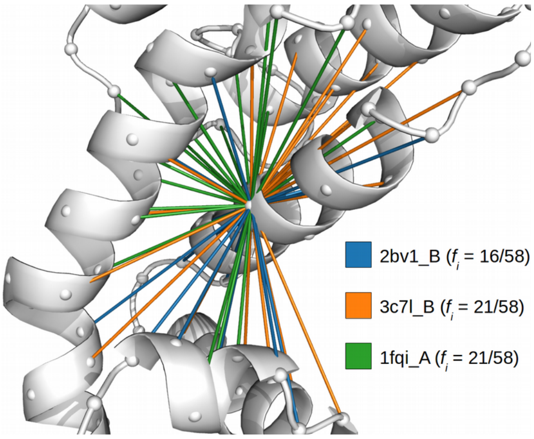

Fig C. Structural detail of the *1dk8_chain_A* model, the same target protein represented in **Fig A** in **S4 Text**. All the 58 Cα-Cα HDDRs acting on residue Trp 45 are represented as lines connecting the Cα of the residue to other Cα atoms. Each HDDR is colored according to its best template, that is, the template with the lowest $|\Delta d_n|$ value for the restrained distance. $f_{i,u}$ values for each template are reported. The figure shows how different best templates are picked at the same time for the same residue and how even in regions close in 3D space, different best templates may be interspersed.

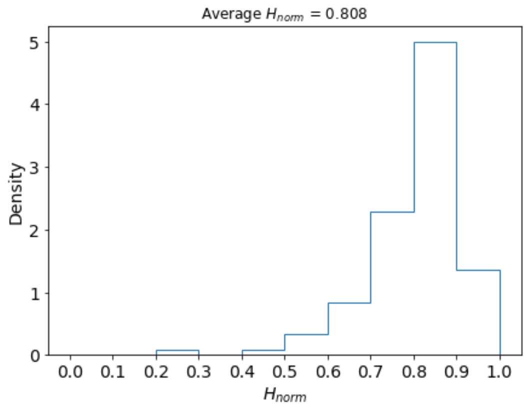

Fig D. Histogram of the H_{norm} values obtained for the 118 models of the AM set.
